# Role of *Waddlia chondrophila* Placental Infection in Miscarriage

**DOI:** 10.3201/eid2003.131019

**Published:** 2014-03

**Authors:** David Baud, Geneviève Goy, Maria-Chiara Osterheld, Antony Croxatto, Nicole Borel, Yvan Vial, Andreas Pospischil, Gilbert Greub

**Affiliations:** University of Lausanne, Lausanne, Switzerland (D. Baud, G. Goy, A. Croxatto, G. Greub);; University Hospital, Lausanne (D. Baud, Y. Vial, G. Greub, M.-C. Osterheld);; University of Zürich, Zürich, Switzerland (N. Borel, A. Pospischil)

**Keywords:** Waddlia chondrophila, Chlamydia-like bacteria, adverse pregnancy outcome, genital tract infection, intracellular bacteria, bacteria, human placenta, miscarriage

## Abstract

*Waddlia chondrophila* is an intracellular bacterium suspected to cause human and bovine abortion. We confirmed an association between antibodies against *W. chondrophila* and human miscarriage and identified this organism in placenta or genital tract of women who had had miscarriages. These results suggest a possible role of *W. chondrophila* infection in miscarriage.

Approximately 25% of pregnant women will experience at least 1 miscarriage (*1*,*2*). However, a cause is identified for only 50% of cases (*3*,*4*). Intracellular bacteria, which do not grow on media routinely used to isolate human pathogens from clinical samples, represent possible agents of miscarriage of unexplained etiology (*4*,*5*).

*Waddlia chondrophila*, a *Chlamydia*-related bacterium first identified in samples of bovine abortion tissues, has been associated with human miscarriages (*6*,*7*). In a study of 438 serum samples from women attending a recurrent-miscarriage clinic, seroprevalence of *W. chondrophila* was higher for women who had sporadic (31.9%) and recurrent (33.0%) miscarriages than for women who had uneventful pregnancies (7.1%; p<0.001) (*6*).

To further investigate the role of *W. chondrophila* in human miscarriage, we studied 386 women who had had miscarriages or uneventful pregnancies. In addition to serologic analysis, we used PCR and immunohistochemical analysis to detect *W. chondrophila* in placenta and vaginal samples.

## The Study

During 2006–2009, a total of 386 women were prospectively enrolled from the obstetrical ward of the University Hospital of Lausanne (Table 1) (*8*). The miscarriage group was composed of 125 women given a diagnosis of an acute episode of miscarriage in the emergency gynecology unit. The control group was composed of 261 women attending a labor ward, having uneventful pregnancies, and having no history of miscarriage, stillbirth, or preterm labor. Age, black race, and number of lifetime sex partners were different between both groups.

**Table 1:** Characteristics of women, by miscarriage history, tested for infection with Waddlia chondrophila*

Characteristic	Control, n = 261	Miscarriage, n = 125	p value†
Age, y, ± SD	31.5 ± 5.0	33.3 ± 6.1	0.002
Race			<0.001	
White	217 (84.8)	69 (71.9)		
Black	20 (7.8)	21 (21.9)		
Asian	19 (7.4)	5 (5.2)		
Other	0	1 (1.0)		
No. lifelong sex partners			0.031	
1	58 (22.2)	37 (29.6)		
2–3	43 (16.5)	24 (19.2)		
4–6	45 (17.2)	10 (8.0)		
>6	36 (13.8)	10 (8.0)		
Unknown	79 (30.3)	44 (35.2)		
*Waddlia spp.–*positive serologic result				
Total Ig ≥64	47 (18.0)	37 (29.6)	0.010	
IgG ≥64	38 (14.6)	29 (23.2)	0.044	
IgM 32	5 (1.9)	1 (0.8)	0.669	
*Waddlia* spp.–positive PCR result				
Vaginal swab specimen	11 (4.2)	9 (7.2)	0.226	
Placenta	11 (4.2)	1 (0.8)	0.113	
*Waddlia *spp.–positive immunohistochemical result	1 (0.4)	2 (0.8)		
*Chlamydia trachomatis s*erologic result				
IgG positive	19 (7.3)	19 (15.2)	0.018	
IgA positive	10 (3.8)	10 (8.0)	0.091	
IgG and IgA positive	7 (2.7)	9 (7.2)	0.037	

*Adapted from Baud et al. (*7,8*). Values are no. (%) unless otherwise indicated. †Statistical analysis was performed only for categorical variables.

Immunofluorescence testing was performed by using *W. chondrophila* as antigen as described (*6*). Eighty-four women had antibodies against *W. chondrophila* as demonstrated by positive immunofluorescence against total immunoglobulin (Table 1). Among them, 67 women had IgG titers ≥1:64 and 6 women had IgM titers ≥1:32 against *W*. *chondrophila* (FluolineG or FluolineM; bioMérieux, Marcy l'Etoile, France). IgG seroprevalence was higher among women who experienced miscarriage (23.2%) than among women who experienced an uneventful pregnancy (14.6%; p = 0.044) (Table 2). When women with and without antibodies against *W. chondrophila* were compared, their age, contact with animals, education, number of previous sex partners, previous contraceptive use, and place of residence (countryside/city) were not associated with a positive serologic result for *W*. *chondrophila*. However, a multivariate logistic regression model indicated that black women were more likely to have antibodies against *W. chondrophila* (odds ratio [OR] 3.15, 95% CI 1.39–7.16).

**Table 2:** Characteristics of 10 women who had had miscarriages and had positive results for *Waddlia chondrophila* by real-time PCR*

Patient no.	Age, y/race	Gravida/parity	No. pregnancy weeks	Total Ig titer	IgG titer	PCR result for vaginal swab specimen	PCR result for placental specimen	Histologic result	IHC result	Other etiology
7	37/white	4/3	11.2	0	0	–	+	No inflammation	–	None found
36	3/7white	1/0	6	64	1,024	–	–	PMN in decidua	–	None found
140	34/black	1/0	6	64	128	–	–	PMN and plasmocytes in decidua compatible with chronic endometritis	–	None found
183	42/white	3/1	9	0	0	+	–	PMN in decidua and glandular epithelium compatible with early infection	–	None found
305	29/white	5/0	21	0	0	+	–	CAM (PMN in chorion and extension of these inflammatory cells to amnios)	–	*Ureaplasma urealyticum* in vaginal swab specimen
357	19/Asian	2/1	8	0	0	+	–	Rare lymphocyte in decidua	–	*Brucella abortus* antibodies
409	42/other	3/1	10	0	0	+	–	PMN in subchorial fibrin and glandular epithelium compatible with early infection	–	HT
459	29/white	3/1	9	0	0	+	–	PMN and hemorrhagic necrosis	–	None found
523	34/other	3/1	10.5	164	0	+	–	No inflammation	+	*Chlamydia trachomatis* antibodies (PCR negative)
535	35/white	3/1	10	164	0	+	–	PMN in fibrin of decidua compatible with early infection	+	None found

*IHC, immunhistochemical; +, positive; –, negative; PMN, polymorphonuclear cells; CAM, chorioamnionitis; HT, hyperthyroidism.

As reported (*8*), we observed an association between miscarriage and *Chlamydia trachomatis* IgG seropositivity. The association between *W. chondrophila* miscarriage and seropositivity remained significant even when adjusted for *C. trachomatis* serostatus and vice versa. In a multivariate logistic regression adjusted for both variables, *C. trachomatis* and *W. chondrophila* seropositivity remained independently associated with miscarriage (OR 2.42, 95% CI 1.22–4.79 and OR 1.87, 95% CI 1.08–3.22, respectively).

After extraction of DNA by using the QIAamp DNA Mini Kit (QIAGEN, Hilden, Germany), we tested all vaginal swab specimens and placenta samples by using a 16S rRNA *Waddlia* spp.–specific real-time PCR as described (*9*). No PCR inhibition was observed. Thirty-two samples (20 vaginal swab specimens and 12 placenta samples) were positive; no sample being positive in both types of samples. Ten of these positive PCR samples were from women who had had miscarriages; 9 of the 10 vaginal swab specimens had a positive PCR result (Tables 1, 2). Two of these 10 patients who had had miscarriages had IgG against *W. chondrophila* (patients 36 and 140). Patient 36 had the highest IgG titer (1,024) of the 386 women. Among the control group, 3 patients had IgG against *W. chondrophila* (titer ≥64). Among these women, 1 had IgG and IgM against *W. chondrophila* and 1 had only IgM against *W. chondrophila* (titer 32).

**Figure 1:**
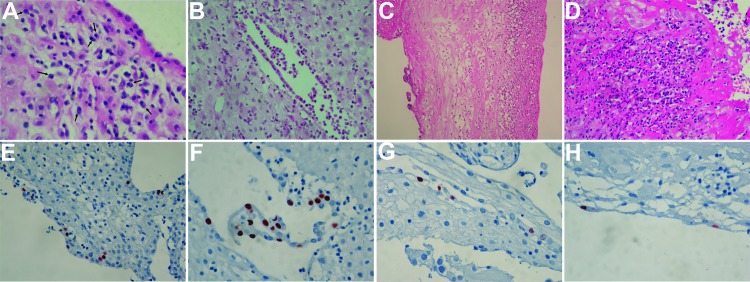
Histopathologic analysis of placentas from women tested for infection with *Waddlia chondrophila*. A) Patient 140, chronic endometritis with various inflammatory cells in the deciduas, including plasmocytes (arrows) (original magnification x600). B, Patient 183, polymorphonuclear cells (PMN) in a an endometrial gland (original magnification x400). C) Patient 305, chorioamnionitis with PMN extending from the chorion to the amnios (original magnification x200). D) Patient 535, PMN in the subchorial fibrin near the gestational sac (original magnification x400). Hematoxylin and eosin stain. Immunohistochemical analysis showing *W. chondrophila* in placental tissue. A rabbit polyclonal antibody directed against *W. chondrophila* was used at a dilution of 1:12,000. Detection was performed by using the ChemMate Kit (Dako, Glostrup, Denmark). Negative controls contained antibody diluent instead of primary antibody. Negative and positive control pellets were included as described (*10*). All highly positive cells were found in epithelium of endometrial glands. E) Patient 535 (miscarriage) (original magnification x400). F) Patient 535 (original magnification x600) G) Patient 523 (miscarriage) (original magnification x600). H) Patient 250 (control) (original magnification x600). 3-amino-9-ethylcarbazole/peroxidase stain and hematoxylin counterstain.

All placenta specimens were examined by a pedopathologist (Table 2; Figure 1, panels A–D). Samples from the 10 women who had miscarriages and positive PCR results showed various histologic features, including deciduitis, chorioamnionitis, and plasmocytes in the decidua, which are compatible with chronic endometritis. Two of the samples showed standard histologic results.

Placentas from the 32 PCR-positive women and 10 PCR-negative controls were tested for *W. chondrophila* by using immunohistochemical analysis with a specific rabbit polyclonal antibody as described (*10*). Three placentas showed positive cells (Table 2; Figure 1, panels E–H). Patients 523 and 535, who had had miscarriages, had positive serologic results for total Ig but negative results for IgG and IgM (Table 2). Patient 250 was a woman who had had an uneventful pregnancy and who had positive PCR results for a vaginal swab specimen but negative serologic results. Immunohistochemical analysis showed that *W. chondrophila* infects mainly cells of the glandular epithelium; *W. chondrophila* was not found in endothelial cells (Figure 1).

**Figure 2:**
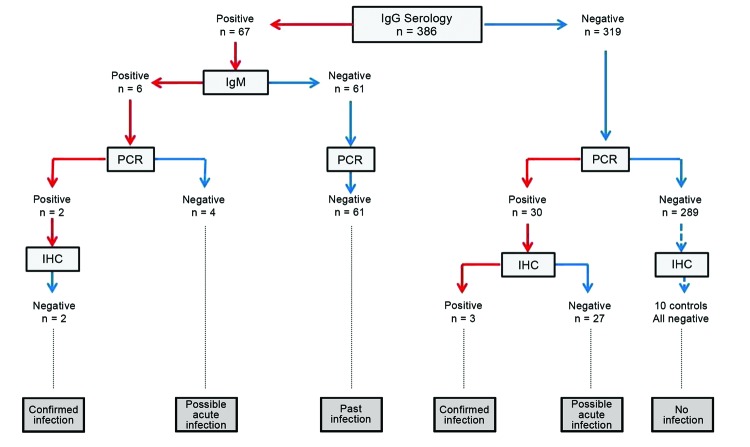
Decision tree for samples from placentas, which were used to screen for infection with *Waddlia chondrophila*. Of 386 women, a total of 5 had a confirmed infection, which was defined as a minimum of 2 independent positive *W. chondrophila*–-specific test results, and 31 had evidence of acute current *W. chondrophila* infection. IHC, immunohistochemical analysis.

Five women showed strong evidence of *W. chondrophila* infection, which was confirmed by ≥2 diagnostic tests (Figure 2). Thus, 2 women who had had a miscarriage had IgM and IgG (titer 32) and positive PCR results. Three other women (2 who had had miscarriages and 1 control) showed positive results by PCR and immunohistochemical analysis. Moreover, 31 other women showed some evidence of acute infection (i.e., 27 with a positive PCR result and/or 4 with IgM against *W. chondrophila*).

## Conclusions

Higher seroprevalence in the miscarriage group confirmed the association between miscarriage and *W. chondrophila* seropositivity observed in a study that investigated a population in London, UK (*6*). We also identified *W. chondrophila* DNA in the placenta and vagina of 32 women, including 10 who had had miscarriages. Among these 10 whose PCR result was positive, 4 were considered as having confirmed cases of infection because they also had positive serologic (n = 2) or immunohistochemical (n = 3) results. *W. chondrophila* in human tissue indicates that this intracellular bacterium might grow or persist within placental cells and might damage the placenta (*11*). The underlying mechanism of *Waddlia*-associated miscarriage may involve bacterial proteins, such as heat-shock protein 60, or production of inflammatory cytokines, such as tumor necrosis factor-α (*5*).

Detection of *W. chondrophila* in the vagina indicates that the infection might have originated after vaginal colonization. However, no association between sexual activity, use of condoms, and positive serologic results for *W. chondrophila* was reported in a study (seroprevalence 8.3%) of 517 young men in Switzerland (*12*).

We identified *W. chondrophila* in the human genital region. However, entry could occur at another site. *W. chondrophila* DNA has also been detected in sputa of patients with pneumonia (*9*,*13*), or other respiratory tract infections could disseminate to the uterus through the bloodstream. In contrast to our previous study findings (*4*), seropositivity for *W. chondrophila* was not associated with contact with animals.

This prospective study confirmed an association between *W. chondrophila* seropositivity and miscarriage. Four (3.2%) of 125 women who had had miscarriages were positive by serologic analysis and PCR or by PCR and immunohistochemical analysis and were considered as having confirmed cases of infection. One (0.4%) *W. chondrophila* infection was documented by 2 diagnostic tests in a women in the control group who had not had a miscarriage (p = 0.04). These results suggest a strong association between *W. chondrophila* infection and miscarriage among women (*6*,*7*). When a *W. chondrophila*–associated miscarriage is suspected, we recommend performing PCR on placenta and vaginal swab specimens and serologic analysis.
